# Uncertainty in banking and debt financing of firms in Vietnam

**DOI:** 10.1371/journal.pone.0305724

**Published:** 2024-07-15

**Authors:** Japan Huynh, Thi Minh Hue Phan

**Affiliations:** Faculty of Finance and Banking, Ho Chi Minh City Open University, Ho Chi Minh City, Vietnam; Universiti Malaysia Sabah, MALAYSIA

## Abstract

This study explores the effects of banking uncertainty on firms’ debt financing. Employing data from 2007 to 2022 of Vietnam–a bank-based economy, we document that banking uncertainty negatively impacts corporate debt. The impact firmly holds across various debt maturities and sources, with the most predominant driver witnessed in bank debt. We also investigate the potential underlying mechanism linking banking uncertainty to debt financing, thereby validating the working of three crucial channels, including increased costs of debt, substitution of trade credit, and contractions in firm investment. Furthermore, conducting extended analysis, we find that debt financing exhibits more pronounced reactions to banking uncertainty for firms with closer ties to banks or during macroeconomic shocks, as captured by the financial crisis and the COVID-19 pandemic. Our findings survive after robustness checks by alternative measurement, static and dynamic econometric models, and endogeneity controls.

## 1. Introduction

The impact of uncertainty stands as a critical topic in the finance literature, reflecting its profound implications for economic and financial activities. Given the inherent uncertainty stemming from policy decision-making and implementation processes, firms may suffer from significant influence on various aspects. The literature reveals that uncertainty manifests its effects across a spectrum of corporate behaviors, encompassing operating performances [1, 2], cash holdings [3–5], capital spending for investment [6–8], earning manipulations [9, 10], dividends [11], corporate innovations [12, 13], corporate default risk [14], and credit spreads [15], among numerous others.

Along this line, the impact of uncertainty on corporate financial structure has been a focal point of extensive research. For example, Zhang et al. [16] demonstrate that increased economic policy uncertainty leads Chinese listed firms to lower their leverage ratios, a finding echoed in diverse contexts by scholars such as Chow et al. [17] and Liu and Zhang [18]. Further, Im et al. [19] identify that uncertainty, proxied by return volatility, significantly influences US firms’ target capital structures, highlighting the overarching impact of uncertainty on corporate financial strategies. This body of work is complemented by Tran and Phan [20] and Fan et al. [21], who explore the nuanced responses of debt contracting and corporate leverage to policy and oil price uncertainties, respectively, showcasing the breadth of uncertainty’s implications. Additionally, Fuller et al. [22] expand the research scope by examining sector-specific impacts and the role of tax uncertainty, broadening our understanding of how uncertainty affects corporate finance. In stark contrast to prior findings, an examination of a sample of Indian-listed firms by Bajaj et al. [23] reveals a positive correlation between economic policy uncertainty and financial leverage. Taken together, these studies offer a broad view of how uncertainty, whether stemming from economic policy, market conditions, or external shocks, shapes firms’ financial strategies across different regions and sectors.

Another substantial body of research underscores the critical role of banking activities in firms’ financial health and investment decisions. Strong relationships with banks significantly enhance firms’ access to financing, especially during economic downturns [24]. The health of the banking sector is equally pivotal, as firms may encounter substantial financing constraints when banks reduce lending [25]. This relationship is particularly vital in emerging markets, where financial systems are often underdeveloped and more volatile than those in advanced economies. Firms in these markets frequently rely on bank financing due to their limited access to capital markets, highlighting the importance of a well-developed banking sector in supporting firm growth and investment [26, 27]. Healthy banks are better equipped to provide stable credit, which is essential for firms to pursue profitable investment opportunities. Conversely, weaker banking systems exacerbate financial constraints, making it more difficult for firms to secure necessary financing [28]. The regulatory environment also plays a crucial role in shaping interactions between banks and firms in emerging markets. Weaker regulatory frameworks in these markets often correlate with higher incidences of banking crises, which can severely limit firms’ financing options and hinder economic development [29, 30]. Overall, the literature highlights the pivotal role of the banking sector in firms’ financial decisions in emerging markets. Further research that sheds light on novel aspects of the bank-firm relationship is essential for understanding and addressing the specific needs of banks and firms operating in these environments.

In this paper, we offer new insights into the effects of uncertainty on corporate financing, from the perspective of banking uncertainty and corporate debt in Vietnam–a bank-based economy. We differentiate this work from previous studies through five aspects discussed as follows. First, in contrast to previous studies focusing on the impact of economic policy uncertainty [16, 18], oil price uncertainty [21], or tax uncertainty [22] on firms’ capital structure, our research provides a novel examination of the effects of uncertainty in banking. The motivation behind our research lies in the acknowledgment that various measures of uncertainty are distinct. Therefore, different uncertainty measures may not capture the same economic and financial shock [31]. This underscores the significance of analyzing multiple uncertainty measures, as each carries unique information. Through our investigation into how banking uncertainty shapes firms’ debt financing, our objective is to enhance comprehension of the intricate interplay between uncertainty and the financial structure of businesses.

Second, as we delve into the effect of banking uncertainty on debt financing of enterprises, we take a more granular approach by dissecting debt holdings into different components based on debt maturity and debt sources. This approach provides deeper insights and a better understanding of the primary drivers of corporate debt and the specific aspects affected by banking uncertainty. Existing literature on the impact of uncertainty on capital structure often overlooks the detailed composition of corporate debt. Consequently, our contribution lies in exploring this aspect from the angles of short-term debt versus long-term debt and bank debt versus other sources of debt, enriching the understanding of how banking uncertainty intricately shapes various dimensions of firms’ debt.

Third, our study thoroughly elucidates the underlying mechanisms by which banking uncertainty influences corporate debt. While existing research acknowledges the impact of uncertainty on firms’ financial structure, it often lacks the identification of the economic mechanisms. Notable exceptions include studies by Fan et al. [21] and Zhang et al. [16]. In detail, Fan et al. [21] posit that oil price uncertainty may decrease corporate leverage by fostering the use of trade credit and escalating the risk of financial distress, while Zhang et al. [16] observe that firms alter their financial structures by incorporating more trade credit during periods of heightened uncertainty. Taking a step further, our paper contends that the relationship between uncertainty and debt financing operates through four primary channels: the cost of debt, trade credit, financial distress, and investment reduction. What sets our approach apart is the comprehensive examination of these four channels within a single study. In this regard, our paper presents another contribution to the literature on the relationship between uncertainty and firms’ financial structure by empirically investigating the comprehensive mechanisms of the effects of banking uncertainty on corporate debt.

Fourth, we explore the interaction of macroeconomic shocks and uncertainty when explaining corporate debt. Existing studies primarily focus on the moderating roles of firm-specific factors, but they often ignore the effect of macroeconomic variables, which might be important for ruling the magnitude of uncertainty and the changes in capital structure. For example, Zhang et al. [16] document that mitigating factors such as regional marketization, state ownership, and prior bank-firm relationships can offset the negative uncertainty effect on leverage ratios. Li and Qiu [32] reveal that the marginal effects of firm characteristics on debt ratios change with economic policy uncertainty. Chow et al. [17] emphasize the moderating role of corporate governance, with board independence and ownership structure influencing the overall effect of uncertainty on leverage decisions. Tran and Phan [20] highlight that government economic policy uncertainty is associated with more stringent debt terms, particularly affecting financially constrained firms. Given the results of these studies, one question that emerges is whether the uncertainty-capital link is heterogeneous across different periods of macroeconomic conditions. In this context, we examine two significant macroeconomic events—the global financial crisis and the coronavirus pandemic. An additional contribution of our study is the novel revelation that these crises moderate the impact of banking-induced uncertainty on corporate debt.

Fifth, we conduct an empirical analysis within the specific context of Vietnam. While some studies have investigated bank uncertainty and corporate behavior in Vietnam [28, 33], there remains a gap in the literature concerning the financing structure of enterprises. As Vietnam is an emerging economy, it is essential to acknowledge its uncertainty influence on financing matters, motivating us to explore the relationship between uncertainty in banking and financing structure of firms and to further recognize an essential channel through which uncertainty may drive the investments and economic development. Notably, the Vietnamese economy is heavily reliant on commercial banks as a pivotal funding source [34, 35]. In other words, the financial condition of Vietnamese firms is intricately tied to the banking system, which they heavily depend on for financial services and funding. This typical relationship creates a mechanism wherein any disruptions or fluctuations in the banking sector exert a direct and substantial influence on firms. Consequently, such disturbances impact the financing access of firms, and this influence is notably more pronounced in shaping overall economic growth in Vietnam compared to other countries. Additionally, Vietnam has undergone an extended period of economic restructuring, and the central bank frequently implements banking reforms due to many important events (such as joining the World Trade Organization in 2007 or the comprehensive structural reform in the banking sector in 2015). As a result, the implementation of these reforms may lead to a significant fluctuation in banking uncertainty.

Examining data from Vietnam during 2007–2022, this study addresses three critical questions: (1) Does banking uncertainty significantly impact corporate debt? (2) What mechanisms connect these two factors? (3) How is the impact moderated under different conditions? Utilizing the cross-sectional dispersion of bank-level shocks to key variables for measuring banking uncertainty, as proposed by Buch et al. [36], we employ static and dynamic panel data estimation approaches to ensure robust results. The findings reveal a notable reduction in corporate debt due to banking uncertainty, affecting both short-term and long-term debt of firms, as well as debt from various sources, with a predominant impact on bank debt. We additionally investigate the potential underlying mechanisms linking banking uncertainty to debt financing using a two-step regression procedure. As a result, our mechanism analysis suggests that banking uncertainty can diminish corporate debt through three crucial channels: escalation in financing costs, amplification in the utilization of trade credit, and curtailment in firm investment. Furthermore, we extend the baseline regression model by introducing interaction terms involving uncertainty with bank-firm relationships and macroeconomic shocks into the analysis. The outcomes indicate that firms with bank-firm relationships experience a heightened negative impact of banking uncertainty on their debt holdings. We also present evidence suggesting that shocks from financial and health crises amplify the adverse effects of banking uncertainty on corporate leverage.

The following sections of the paper are organized as outlined below. Section 2 provides a literature review, laying the groundwork for our predictions regarding the impacts of banking uncertainty on debt financing. Section 3 outlines the sample selection, variable measurement, and model specifications. Section 4 presents the empirical findings, encompassing the baseline association, mechanism tests, and robustness checks. Then, Section 5 addresses the extended analysis, and Section 6 concludes the work.

## 2. Literature review and hypotheses development

The literature has commonly implied a negative association between uncertainty and corporate debt, elucidating at least four underlying mechanisms: the cost of debt, trade credit, financial distress, and investment contractions.

Concerning the first channel, theoretical models posit that heightened uncertainty amplifies corporate borrowing costs. This prompts firms to decrease leverage as a strategic measure to uphold financial stability and pursue financial flexibility. The rationale behind this approach lies in the potential apprehensions of creditors regarding the adverse impact of uncertainty on the payment capacity of borrowers; in response, lenders may impose higher risk premiums as compensation for the augmented lending risk amid elevated uncertainty [37]. Furthermore, the constriction of credit supply is a widely recognized outcome of general uncertainty [38, 39] and even banking uncertainty [34, 36]. In an effort to manage risks and avert financial instability, banks reduce loan supply by elevating interest rates, thereby escalating the cost of debt.

In the context of the second channel, Petersen and Rajan [40] propose a hypothesis asserting that in periods characterized by heightened economic uncertainty, suppliers assume the role of lenders of last resort for financially constrained customers. This implies a surge in accounts payable during uncertain periods. Additionally, firms engaging with customers possessing significant market power are inclined to extend more trade credit in times of uncertainty, as the potential loss of sales could significantly impact their financial stability [41]. Apart from depending on loans from financial institutions, trade credit functions as a vital informal financing avenue, especially for businesses in developing markets [42]. Hence, firms widely resort to trade credit as a substitute for bank loans to fulfill their financing requirements. In accordance with these perspectives, the heightened utilization of trade credit may contribute to a reduction in debt financing.

For the third channel, uncertainty has the potential to drive a firm towards lower leverage by intensifying financial distress. Under conditions of uncertainty, firm earnings exhibit heightened volatility [7]. Building upon these arguments, it can be asserted that uncertainty may render firms’ market value more precarious and volatile, contributing to increased uncertainty surrounding future cash flows, consequently elevating default risk and financial distress. Accordingly, the augmented risk of financial distress will likely result in decreased corporate leverage.

Regarding the fourth channel, firms exhibit a reduction in their financing demands in response to escalating uncertainty. Previous research establishes that firms adopt a more conservative approach in making investment decisions in the presence of high uncertainty, influenced by investment irreversibility under the real option theory [43]. Many empirical studies support this notion, indicating that uncertainty contributes to a decrease in investments [6–8]. These contractions in investment have a detrimental effect on firms’ inclination to employ more debt.

Drawing on the aforementioned arguments, it can be inferred that there exists a negative correlation between uncertainty and corporate debt, which has also been widely evidenced in the literature across various forms of uncertainty [16–18, 20–22]. Consequently, we posit the following hypothesis:

*Hypothesis A*: *Banking uncertainty has a negative effect on debt financing of firms*.

Nevertheless, some studies propose that uncertainty may not consistently diminish corporate debt. The escalation of uncertainty heightens the unpredictability of anticipated earnings for firms [7]; consequently, firms may turn to external sources of finance to navigate through periods of business uncertainty. As previously posited, the cost of debt tends to surge during uncertain times. According to fundamental principles in finance, the cost of debt should theoretically be lower than the cost of equity due to its lower risk profile (attributed to fixed interest obligations, priority in dividend payments and liquidation, and associated tax shields). Given this, the cost of debt experiences an increase in times of uncertainty, albeit remaining comparatively lower than the cost of equity [44]. This prompts firms to favor debt over equity, resulting in heightened leverage amidst uncertainty. Notably, Bajaj et al. [23] substantiate this prediction by analyzing capital structure under the dynamic trade-off theory, revealing a positive long-term association between economic uncertainty and leverage.

Besides, during periods of banking uncertainty, financial institutions may become more risk-averse and tighten their lending criteria [45]. Consequently, firms might increase their borrowing to secure liquidity in anticipation of future credit constraints, and this preemptive borrowing can lead to an overall rise in corporate debt [25]. Similarly, uncertainty in the banking sector often leads to fluctuations in interest rates. Companies may take on more debt to lock in current rates, fearing that borrowing costs could rise further. This is particularly true for firms that rely heavily on external financing. Accordingly, we hypothesize that:

*Hypothesis B*: *Banking uncertainty has a positive effect on debt financing of firms*.

Amid conflicting literature and divergent findings, the present study makes a valuable contribution to the existing body of literature by investigating the impact of banking uncertainty on firms’ debt holdings. Notably, this study advances our comprehension of the various channels through which these effects manifest.

## 3. Empirical design

### 3.1. Banking uncertainty measurement

This paper captures uncertainty using the method of Buch et al. [36] that fits well to exhibit disaggregate uncertainty in the banking system. As suggested by these earlier researchers, their banking uncertainty index operates on the principle that the predictability of future outcomes diminishes with rising uncertainty; hence, an expanded distribution of shocks to vital bank-level variables signifies heightened uncertainty within the banking system. By bank-level data, Buch et al. [36] introduce the following equation to calculate the bank-year-specific shocks of key bank-level variables:

$$X_{i,t}=\alpha_{i}+\beta_{t}+\varepsilon_{i,t\;}$$ (1)

in which *X*_*i*,*t*_ denotes the key bank-level variable in year *t* for bank *i*, including the asset growth, the funding growth and the return-to-asset ratio, separately. While conducting the analysis, we utilize the dispersion of shocks to all these three variables as a robustness test. Eq (1) also displays bank fixed effects (*α*_*i*_) and time fixed effects (*β*_*t*_). In this stream, bank-year-specific shocks are shown in the form of the residuals *ε*_*i*,*t*_, which we need to collect to compute their dispersion to yield our uncertainty measure for the banking system in year *t* as follows:

$$Uncertainty_{t}=SD\left(\varepsilon_{i,t}\right)$$ (2)

With Eq (2), the standard deviation of the residuals *SD*(*ε*_*i*,*t*_) is employed, and its larger outcome indicates a higher degree of uncertainty in banking. According to Buch et al. [36], in case banks can approach the type of information reflected by bank and time fixed effects, and assuming that such information is taken advantage of by banks, this measure here could capture the uncertainty perceived by banks. From a technical standpoint, this approach shares the same spirit with Bloom et al. [46], who display that the cross-sectional dispersion based on firm-level data of US manufacturing firms can reveal variations in movements of business cycles. Lately, the methodology outlined above for constructing such an uncertainty metric for the banking system has gained extensive traction in financial research [1, 28, 47, 48].

### 3.2. Model specifications

We investigate the influence of banking uncertainty on corporate debt via the following foundational empirical model:

$$Debt_{i,t}=\alpha_{0}+\alpha_{1}\times Uncertainty_{t-1}+\beta \times Firm_{i,t-1}+\gamma \times Macro_{t-1}+\alpha_{i}+\varepsilon_{i,t\;}$$ (3)

where subscripts *i* and *t* correspond to firms and years, respectively. The dependent variable is employed to capture corporate debt, specifically defined as the total debt scaled by total assets (*debt1*). Additionally, we employ another measure to delve into the intricacies of the firm’s debt structure, i.e., the ratio of total debt to total liabilities (*debt2*). For the independent variable of primary interest, *Uncertainty*, we utilize the previously designed annual and continuous index of banking uncertainty. To further explore the impact of banking uncertainty on debt nature and maturity, we construct two sets of decomposed variables. These include (i) the ratio of short-term debt to total assets (*shortdebt*) and the ratio of long-term debt to total assets (*longdebt*), and (ii) bank debt divided by total assets (*bankdebt*) and other debt scaled by total assets (*otherdebt*).

Adhering to the financial structure literature [17, 18, 21, 22], we incorporate a comprehensive array of firm control variables (*Firm*), encompassing firm size, sales, tangibility, return, and state ownership. Recognizing that firms’ financing decisions may evolve with changing macroeconomic conditions, we additionally integrate economic cycles and monetary policy, reflected in the vector *Macro* of our model. Detailed definitions of these critical variables are outlined in Table 1. Firm fixed effects, denoted as *α*_*i*_, encapsulate the individual characteristics of firms that remain constant over time, which is also helpful to mitigate the impact of omitted variables. The error term in our model is represented by *ε*_*i*,*t*_. We lag the independent variables to address potential reverse-causality concerns and consider firms’ delayed responses to uncertainty and other shocks [47, 49–52]. It should also be noted that we examined the effects of both non-lagged uncertainty values and those with two-period (and beyond) lags; however, these results were consistently insignificant. Therefore, our paper focuses solely on the findings from one-period lagged uncertainty.

**Table 1:** Descriptive statistics.

	Mean	SD	Min	Max	Definition
debt1	0,18	0,18	0,00	0,65	Total interest-bearing debt divided by total assets
debt2	0,34	0,29	0,00	0,90	Total interest-bearing debt divided by total liabilities
shortdebt	0,12	0,15	0,00	0,57	Short-term debt divided by total assets
longdebt	0,06	0,11	0,00	0,51	Long-term debt divided by total assets
bankdebt	0,17	0,18	0,00	0,64	Bank debt divided by total assets
otherdebt	0,01	0,03	0,00	0,22	Other debt divided by total assets
aunc	0,28	0,37	0,11	2,14	Banking uncertainty, estimated by the dispersion of asset shocks
func	0,39	0,47	0,10	2,25	Banking uncertainty, estimated by the dispersion of fund shocks
runc	0,01	0,00	0,00	0,01	Banking uncertainty, estimated by the dispersion of return shocks
size	27,15	1,59	23,74	31,52	Natural logarithm of total assets
sales	0,27	0,96	-0,76	7,41	Sales growth rate
ppe	0,18	0,18	0,00	0,79	Tangible fixed assets divided by total assets
roa	0,06	0,07	-0,12	0,33	Net return on assets
sof	0,25	0,44	0,00	1,00	A dummy variable that equals 1 when the state possesses more than 50% of the shares of the firms, and 0 otherwise (in line with the Law on Enterprises in Vietnam)
refinance	0,07	0,03	0,04	0,15	Refinancing rates set up by the central bank
gdp	0,06	0,02	0,03	0,08	GDP growth rate

Initially, we estimate Eq (3) employing firm fixed effects and robust Driscoll-Kraay standard errors, effectively accommodating cross-sectional dependence [53]. Subsequently, to address endogeneity concerns and account for the dynamic nature of corporate debt financing behavior, we employ the two-step system generalized method of moments (GMM) estimator. This approach incorporates lagged levels and differences as valid instruments within a single system, thereby addressing unobserved heterogeneity and simultaneity [54]. Some specification tests are conducted to validate the reliability of the GMM model, including AR(1)/AR(2) tests for first and second-order autocorrelation of the residuals and the Hansen test of jointly valid instruments.

### 3.3. Data and descriptive statistics

The data employed in this study comprises two primary components: corporate financial information and bank data. Our corporate sample consists of publicly listed firms on the Ho Chi Minh City (HOSE) and Hanoi (HNX) stock exchanges, excluding financial entities such as banks and insurance companies and firms with missing data necessary for computing research variables. Concerning bank-level information, we approach all commercial banks in Vietnam with available financial data, as other forms of banks (e.g., policy banks) represent a minor portion of the banking system and operate under different business regimes. Subject to the data availability, this study spans the period from 2007 to 2022, with data sourced from the FiinPro database. As a result, we have data from 40 banks with 534 observations to estimate banking uncertainty, which makes up over 99% of the banking market shares in Vietnam, and a final sample containing 623 firms with 7,592 firm-year observations for regression analysis. Concurrently, for macro variables, we retrieve yearly data on GDP and policy rates from the World Development Indicators database.

Table 1 provides the descriptive statistics of the variables. To mitigate the impact of outliers, we employ winsorization of the 1st/99th percentiles for continuous firm-level variables. Notably, total debt constitutes approximately 18% of total assets from 2007 to 2022, spanning from 0% to 65%. Intriguingly, debt comprises roughly a third of a business’s total liabilities, suggesting that Vietnamese firms still employ alternative means to finance their activities. A more granular examination reveals a higher utilization of short-term debt than long-term debt, with bank debt accounting for 17% of total assets and constituting a significant proportion of total interest-bearing debt. Overall, the debt ratio of Vietnamese firms has tended to narrow in recent years, converging toward patterns observed in developed countries. This trend may be attributed to the sample composition of listed firms and the rapid development of the stock market. However, the prevalence of bank debt remains pronounced when firms need external financing, thereby once again justifying the motivation to conduct the present research in Vietnam.

Additionally, the level of banking uncertainty in Vietnam displays significant variation, particularly evident in the measures of *aunc* and *func*, while the *runc* variable exhibits a distinct pattern with minimal fluctuation over the same period (with standard deviation only standing at 0.002). Thus, we decide not to use this *runc* variable when conducting regressions, in line with the banking uncertainty literature [28]. Next, correlation coefficients for the variables are computed (not reported for brevity). It is important to note that all correlation coefficients between explanatory variables generally fall below 0.4, indicating that the multicollinearity problems is not a concern.

## 4. Empirical results

## 4.1. Baseline regression results

This subsection highlights the outcomes from the baseline empirical analyses. Initially, we conduct estimations using the fixed effect model, and the results are presented in Table 2. Then, Table 3 presents the results of our GMM model with internal instruments. In these GMM estimations, we indicate the joint validity of the instruments and the absence of second-order autocorrelation. Besides, the coefficient for the lagged dependent variable is found to be statistically significant and negative, suggesting that firms with higher debt ratios tend to employ lower debt ratios in the subsequent period.

**Table 2:** Banking uncertainty and corporate debt: Baseline fixed effect regression results.

	(1)	(2)	(3)	(4)
	debt1	debt1	debt2	debt2
aunc	-0.151***		-0.224***	
	(0.021)		(0.033)	
func		-0.050***		-0.075***
		(0.008)		(0.014)
size	0.034***	0.033***	0.041***	0.040***
	(0.004)	(0.004)	(0.006)	(0.007)
sales	0.005**	0.006**	0.008*	0.008**
	(0.002)	(0.002)	(0.004)	(0.004)
ppe	0.014	0.015	0.094***	0.096***
	(0.015)	(0.015)	(0.024)	(0.024)
roa	-0.279***	-0.266***	-0.326***	-0.306***
	(0.028)	(0.024)	(0.062)	(0.054)
sof	-0.004	-0.005	-0.010	-0.012
	(0.007)	(0.007)	(0.011)	(0.012)
refinance	0.474*	0.429	0.618	0.549
	(0.244)	(0.249)	(0.392)	(0.406)
gdp	0.985**	1.124***	1.617**	1.826**
	(0.344)	(0.373)	(0.590)	(0.633)
Observations	6,969	6,969	6,969	6,969
Firms	623	623	623	623
R-squared	0.066	0.063	0.051	0.049

Notes: Dependent variables (DV) are shown at the top.

* p<0.1;

** p<0.05;

*** p<0.01.

Standard errors are in parentheses.

**Table 3:** Banking uncertainty and corporate debt: Baseline GMM regression results.

	(1)	(2)	(3)	(4)
	debt1	debt1	debt2	debt2
aunc	-0.160***		-0.277***	
	(0.011)		(0.017)	
func		-0.053***		-0.095***
		(0.004)		(0.006)
size	0.038***	0.037***	0.057***	0.056***
	(0.003)	(0.003)	(0.004)	(0.004)
sales	0.002**	0.003**	0.003	0.003
	(0.001)	(0.001)	(0.002)	(0.002)
ppe	0.139***	0.136***	0.319***	0.316***
	(0.019)	(0.019)	(0.028)	(0.028)
roa	-0.514***	-0.500***	-0.594***	-0.565***
	(0.038)	(0.038)	(0.062)	(0.062)
sof	-0.008	-0.011	-0.053***	-0.056***
	(0.008)	(0.008)	(0.012)	(0.012)
refinance	0.465***	0.419***	0.662***	0.539***
	(0.044)	(0.046)	(0.071)	(0.075)
gdp	0.723***	0.905***	1.257***	1.585***
	(0.083)	(0.085)	(0.156)	(0.159)
lagged DV	-0.048***	-0.041**	-0.048***	-0.040**
	(0.017)	(0.017)	(0.017)	(0.017)
Observations	6,969	6,969	6,969	6,969
Firms	623	623	623	623
Instruments	120	120	120	120
AR(1) test	0.000	0.000	0.000	0.000
AR(2) test	0.462	0.128	0.114	0.123
Hansen test	0.520	0.203	0.245	0.412

Notes: Dependent variables (DV) are shown at the top.

* p<0.1;

** p<0.05;

*** p<0.01.

Standard errors are in parentheses.

Across all estimations in two tables, we observe a significantly negative association between banking uncertainty and corporate debt at the 1% level. The effect is not only statistically significant but also economically substantial. For instance, a one standard deviation increase in the uncertainty of *aunc* (0.37) may lead to a reduction in the debt ratio by 0.056 points (0.151*0.37, as taken from column 1 of Table 2), constituting approximately 31% of the sample mean of *debt1* (0.18). This pattern persists across different variants of banking uncertainty and debt ratios and for both static and dynamic models. Therefore, our findings align with Hypothesis A, indicating that uncertainty prompts firms to decrease their reliance on debt financing. This result is also consistent with prior empirical studies examining other forms of uncertainty [16–18, 20–22].

Regarding control variables, the coefficient for firm size is significantly positive, indicating that larger firms tend to utilize more debt. This pattern aligns with the understanding that larger firms, endowed with better reputation and diversification, face a lower probability of bankruptcy, thereby enhancing their propensity to employ debt. We also find that sales growth is positively correlated with the debt ratio, supporting the idea that firms with substantial growth opportunities predominantly rely on debt to finance their investments. Next, the significantly positive association between tangibility and debt aligns with the perspective that firms possessing greater tangibility have more assets available as collateral for bank loans, facilitating easier access to debt funding. Profitability exhibits a significant negative correlation with the debt ratio, consistent with the pecking order theory. Interestingly, we observe some evidence indicating that state-owned enterprises exhibit lower debt buffers. In general, these findings concerning firm controls are in agreement with the studies by Zhang et al. [16] and Chow et al. [17]. Additionally, we note that firms’ debt financing undergoes certain changes in response to macro-environmental fluctuations, as reflected through economic cycles and monetary policy.

### 4.2. Decomposing corporate debt

We conduct a re-estimation of the baseline regression model, incorporating short-term and long-term debt, to further explore the distinct effects of banking uncertainty on the maturity structure of debt. This analysis is prompted by some critical considerations. Precisely, the debt maturity design of firms, involving the choice between short-term and long-term debt, constitutes a pivotal financing decision. From the firms’ perspective, shortening maturity elevates refinancing risk and exposes them to higher liquidity and underinvestment risk [55]. On the side of banks, during periods marked by elevated banking uncertainty, there may be a tendency to grant shorter-term credit rather than longer-term loans, given that lending short-term debt poses less risk for them [7]. Therefore, the reduction in long-term debt could be more pronounced. Given these rationales, it is intriguing to investigate how banking volatility influences firms’ access to short-term versus long-term debt.

We replicate our estimations by including newly decomposed variables and present the outcomes in Table 4. The results indicate a significantly negative relationship between uncertainty and both measures of debt. However, considering the magnitude of the coefficients, we fail to discern a consistent pattern regarding which maturity effect is dominant. Our findings complement those of Hasan et al. [56], who illustrate that oil price uncertainty is associated with a higher prevalence of short-term debt compared to long-term debt.

**Table 4:** Decomposing corporate debt: Long-term versus short-term debt.

	Fixed effect regressions (columns 1–4)				GMM regressions (columns 5–8)			
	(1)	(2)	(3)	(4)	(5)	(6)	(7)	(8)
	shortdebt	shortdebt	longdebt	longdebt	shortdebt	shortdebt	longdebt	longdebt
aunc	-0.037***		-0.112***		-0.127***		-0.050***	
	(0.009)		(0.014)		(0.012)		(0.006)	
func		-0.010**		-0.040***		-0.044***		-0.019***
		(0.005)		(0.004)		(0.004)		(0.002)
size	0.008**	0.007**	0.026***	0.025***	0.017***	0.017***	0.014***	0.014***
	(0.003)	(0.003)	(0.002)	(0.003)	(0.003)	(0.003)	(0.001)	(0.001)
sales	0.003***	0.003***	0.003	0.003*	-0.001	-0.001	0.003***	0.003***
	(0.001)	(0.001)	(0.002)	(0.002)	(0.001)	(0.001)	(0.001)	(0.001)
ppe	0.005	0.005	0.013	0.014	0.011	0.012	0.096***	0.095***
	(0.011)	(0.011)	(0.009)	(0.009)	(0.019)	(0.019)	(0.009)	(0.009)
roa	-0.145***	-0.140***	-0.132***	-0.124***	-0.287***	-0.284***	-0.104***	-0.105***
	(0.014)	(0.013)	(0.018)	(0.017)	(0.031)	(0.031)	(0.015)	(0.015)
sof	-0.003	-0.003	-0.002	-0.003	-0.019**	-0.020**	-0.002	-0.001
	(0.005)	(0.005)	(0.003)	(0.003)	(0.008)	(0.008)	(0.003)	(0.003)
refinance	0.239**	0.239*	0.226	0.183	0.239***	0.165***	0.097***	0.068***
	(0.108)	(0.114)	(0.149)	(0.143)	(0.043)	(0.047)	(0.019)	(0.020)
gdp	0.724***	0.748***	0.244	0.358*	0.493***	0.657***	0.114***	0.178***
	(0.203)	(0.204)	(0.168)	(0.198)	(0.069)	(0.074)	(0.028)	(0.029)
lagged DV					-0.076***	-0.077***	0.141***	0.145***
					(0.018)	(0.018)	(0.015)	(0.015)
Observations	6,969	6,969	6,969	6,969	6,969	6,969	6,969	6,969
Firms	623	623	623	623	623	623	623	623
R-squared	0.030	0.029	0.057	0.054				
Instruments					120	120	120	120
AR(1) test					0.000	0.000	0.000	0.000
AR(2) test					0.220	0.197	0.811	0.954
Hansen test					0.234	0.267	0.175	0.133

Notes: Dependent variables (DV) are shown at the top.

* p<0.1;

** p<0.05;

*** p<0.01.

Standard errors are in parentheses.

Additionally, in line with the study’s focus on the impact of banking uncertainty on firms’ financing, a specific examination of how firms’ ownership of bank debt is affected becomes pertinent. To provide additional insights into this aspect, we decompose the total debt of firms into bank debt and debt from other sources, such as bond issuance. The outcomes of this decomposition, as presented in Table 5, reveal that uncertainty diminishes different forms of debt, with a notably more substantial effect observed on bank debt. However, this result can be attributed to the much larger proportion of bank debt within firms’ overall debt portfolios.

**Table 5:** Decomposing corporate debt: Bank debt versus other debts.

	Fixed effect regressions (columns 1–4)				GMM regressions (columns 5–8)			
	(1)	(2)	(3)	(4)	(5)	(6)	(7)	(8)
	bankdebt	bankdebt	otherdebt	otherdebt	bankdebt	bankdebt	otherdebt	otherdebt
aunc	-0.124***		-0.024***		-0.136***		-0.007***	
	(0.020)		(0.003)		(0.011)		(0.002)	
func		-0.039***		-0.009***		-0.044***		-0.003***
		(0.008)		(0.001)		(0.004)		(0.001)
size	0.028***	0.026***	0.006***	0.006***	0.032***	0.032***	0.002***	0.002***
	(0.004)	(0.005)	(0.001)	(0.001)	(0.003)	(0.003)	(0.001)	(0.001)
sales	0.005***	0.005***	0.001	0.001	0.002	0.002	0.001***	0.001*
	(0.002)	(0.002)	(0.001)	(0.001)	(0.001)	(0.001)	(0.001)	(0.001)
ppe	0.023	0.024	-0.002	-0.002	0.149***	0.147***	-0.001	-0.001
	(0.016)	(0.016)	(0.004)	(0.004)	(0.019)	(0.019)	(0.002)	(0.002)
roa	-0.259***	-0.247***	-0.017***	-0.016***	-0.466***	-0.451***	-0.010***	-0.009***
	(0.025)	(0.023)	(0.005)	(0.004)	(0.037)	(0.036)	(0.003)	(0.003)
sof	-0.004	-0.004	-0.001	-0.001	-0.003	-0.005	-0.002***	-0.002***
	(0.007)	(0.007)	(0.001)	(0.001)	(0.008)	(0.008)	(0.001)	(0.001)
refinance	0.388*	0.361	0.083**	0.070**	0.385***	0.349***	0.027***	0.023***
	(0.218)	(0.229)	(0.032)	(0.026)	(0.045)	(0.047)	(0.005)	(0.005)
gdp	1.109***	1.214***	-0.107***	-0.079**	0.773***	0.924***	-0.039***	-0.033***
	(0.345)	(0.367)	(0.028)	(0.029)	(0.083)	(0.085)	(0.008)	(0.007)
lagged DV					-0.045**	-0.037**	0.061***	0.059***
					(0.018)	(0.018)	(0.011)	(0.011)
Observations	6,969	6,969	6,969	6,969	6,969	6,969	6,969	6,969
Firms	623	623	623	623	623	623	623	623
R-squared	0.061	0.058	0.082	0.087				
Instruments					120	120	120	120
AR(1) test					0.000	0.000	0.000	0.000
AR(2) test					0.153	0.148	0.810	0.785
Hansen test					0.411	0.388	0.122	0.112

Notes: Dependent variables (DV) are shown at the top.

* p<0.1;

** p<0.05;

*** p<0.01.

Standard errors are in parentheses.

Collectively, the results from our in-depth regressions add more value to our work. They affirm that banking uncertainty exerts adverse effects on the firm’s debt buffers, irrespective of debt maturity and nature.

### 4.3. Some robustness tests

#### 4.3.1. The growth rate of debt

In an effort to delve deeper into our findings, we employ an alternative dimension for the dependent variable. One might argue that the ratios of debt to total assets or liabilities may not fully capture how firms adjust their debt holdings in response to heightened uncertainty, as these ratios are more indicative of financial structure. In other words, they fail to assess the shift in the volume of debt. To address this concern, we incorporate the growth rate of debt as an alternative measure, which helps to indicate how firms expand their holdings of debt. We re-run the baseline model with the growth rate of debt as the dependent variable, and the results are presented in Table 6. Notably, we continue to have evidence in favor of a drop in corporate debt under the pressure of banking uncertainty.

**Table 6:** Robustness tests: A look into the growth rate of corporate debt.

	Fixed effect regressions (columns 1–2)		GMM regressions (columns 3–4)	
	(1)	(2)	(3)	(4)
	debtgrowth	debtgrowth	debtgrowth	debtgrowth
aunc	-0.180***		-0.303***	
	(0.031)		(0.022)	
func		-0.058***		-0.100***
		(0.014)		(0.008)
size	0.030***	0.029***	0.053***	0.052***
	(0.007)	(0.008)	(0.005)	(0.005)
sales	0.009**	0.009**	0.003	0.003
	(0.003)	(0.003)	(0.002)	(0.002)
ppe	0.107***	0.108***	0.341***	0.340***
	(0.027)	(0.027)	(0.030)	(0.030)
roa	-0.309***	-0.292***	-0.598***	-0.577***
	(0.056)	(0.051)	(0.066)	(0.065)
sof	-0.009	-0.010	-0.057***	-0.059***
	(0.011)	(0.012)	(0.013)	(0.013)
refinance	0.470	0.426	0.438***	0.294***
	(0.354)	(0.376)	(0.086)	(0.091)
gdp	1.836***	1.992***	1.303***	1.644***
	(0.589)	(0.619)	(0.180)	(0.185)
lagged DV			-0.088***	-0.081***
			(0.022)	(0.022)
Observations	6,969	6,969	6,969	6,969
Firms	623	623	623	623
R-squared	0.048	0.046		
Instruments			73	73
AR(1) test			0.000	0.000
AR(2) test			0.104	0.135
Hansen test			0.102	0.103

Notes: Dependent variables (DV) are shown at the top.

* p<0.1;

** p<0.05;

*** p<0.01.

Standard errors are in parentheses.

#### 4.3.2. Industry fixed effects

We also enhance the robustness of our results by adjusting for fixed effects. Due to differences in financing structures across industries, it is necessary to include industry fixed effects in our estimations. Consequently, we incorporate industry fixed effects into the model, replacing firm fixed effects, and re-estimate the baseline regression using the dynamic GMM estimator. The results presented in Table 7 demonstrate that the impact of banking uncertainty on firms’ debt levels remains consistent across all models.

**Table 7:** Robustness tests: Estimations with industry fixed effects.

	(1)	(2)	(3)	(4)
	debt1	debt1	debt2	debt2
aunc	-0.109***		-0.191***	
	(0.012)		(0.020)	
func		-0.035***		-0.068***
		(0.004)		(0.007)
size	0.001	-0.001	-0.006	-0.009
	(0.006)	(0.006)	(0.011)	(0.011)
sales	0.000	0.001	0.001	0.001
	(0.002)	(0.002)	(0.003)	(0.003)
ppe	-0.091*	-0.101*	0.119	0.119
	(0.055)	(0.056)	(0.082)	(0.084)
roa	-0.505***	-0.507***	-0.438***	-0.447***
	(0.085)	(0.085)	(0.124)	(0.126)
sof	-0.003	-0.003	-0.096***	-0.113***
	(0.022)	(0.023)	(0.037)	(0.039)
refinance	0.278***	0.246***	0.357***	0.245***
	(0.048)	(0.050)	(0.078)	(0.083)
gdp	0.631***	0.752***	1.039***	1.282***
	(0.097)	(0.102)	(0.161)	(0.171)
lagged DV	-0.059***	-0.055***	-0.060***	-0.057***
	(0.018)	(0.018)	(0.017)	(0.017)
Observations	6,969	6,969	6,969	6,969
Firms	623	623	623	623
Industry fixed effects	Yes	Yes	Yes	Yes
Instruments	120	120	120	120
AR(1) test	0.000	0.000	0.000	0.000
AR(2) test	0.267	0.239	0.228	0.234
Hansen test	0.298	0.311	0.431	0.402

Notes: Dependent variables (DV) are shown at the top.

* p<0.1;

*** p<0.01.

Standard errors are in parentheses.

#### 4.3.3. Further controlling for endogeneity

Endogeneity poses a great challenge in accurately identifying the effect of uncertainty on debt ratios. While we employ several techniques to mitigate endogeneity, such as incorporating firm fixed effects, including lagged variables, and employing GMM estimations, it is likely that potential endogeneity concerns persist. To comprehensively address these concerns, we adopt instrumental variables (IV) and implement a two-stage least squares (2SLS) estimation procedure.

For this purpose, we employ the Economic Policy Uncertainty (EPU) index of China (Vietnam’s largest trading partner) as an instrumental variable in the IV-2SLS regression. This instrumental variable is considered fitted as it may directly impact Vietnamese uncertainty, yet there is no evident proof to suggest a direct impact on the financing of Vietnamese firms. The EPU data are sourced in monthly form from https://www.policyuncertainty.com website, which needs to be converted into annual average values to match our available panel data. Next, inspired by Fungáčová et al. [57], we propose that the past performance of the banking sector in Vietnam may be correlated with the level of current banking uncertainty, but not directly with the current financing of firms. Therefore, to construct an alternative instrumental variable, we collect data on bank performance with a five-year lag from the World Development Indicators database [57].

We then perform two stages of regressions. Indeed, first-stage results (not reported for brevity) justify the use of our instruments and final results from the second stage as presented in Table 8 consistently show a negative relationship between banking uncertainty and firm debt. Hence, the additional tests conducted under the IV-2SLS approach provide further confirmation of our main findings.

**Table 8:** Robustness tests: Addressing endogeneity with IV-2SLS regressions.

	Instrument: EPU China (columns 1–4)				Instrument: Lagged banking performance (columns 5–8)			
	(1)	(2)	(3)	(4)	(5)	(6)	(7)	(8)
	debt1	debt1	debt2	debt2	debt1	debt1	debt2	debt2
aunc	-1.585***		-2.666***		-1.143***		-1.977***	
	(0.158)		(0.269)		(0.252)		(0.428)	
func		-2.135***		-3.592***		-0.086***		-0.148***
		(0.648)		(1.093)		(0.013)		(0.023)
size	0.133***	0.418***	0.210***	0.689***	0.055***	0.008*	0.110***	0.001
	(0.012)	(0.120)	(0.020)	(0.203)	(0.018)	(0.004)	(0.030)	(0.007)
sales	0.006**	0.037**	0.011**	0.063**	0.016***	0.008***	0.025***	0.013***
	(0.003)	(0.015)	(0.005)	(0.026)	(0.003)	(0.002)	(0.005)	(0.003)
ppe	0.050*	0.226**	0.016	0.311*	0.072***	0.031*	0.193***	0.122***
	(0.027)	(0.109)	(0.046)	(0.184)	(0.027)	(0.017)	(0.046)	(0.029)
roa	-0.747***	-1.757***	-1.121***	-2.821***	0.142	-0.169***	-0.392**	-0.147***
	(0.070)	(0.485)	(0.119)	(0.818)	(0.093)	(0.033)	(0.159)	(0.056)
sof	-0.021**	-0.122***	-0.039**	-0.208***	0.011	0.002	0.015	0.001
	(0.010)	(0.047)	(0.017)	(0.079)	(0.010)	(0.007)	(0.017)	(0.011)
refinance	-1.233***	-8.870***	-2.287***	-15.136***	2.012***	1.034***	3.237***	1.545***
	(0.211)	(2.904)	(0.359)	(4.899)	(0.313)	(0.087)	(0.532)	(0.148)
gdp	1.867***	10.725***	3.119***	18.021***	0.190	0.499***	0.264	0.798***
	(0.186)	(3.021)	(0.316)	(5.097)	(0.215)	(0.122)	(0.365)	(0.207)
Observations	6,958	6,958	6,958	6,958	6,958	6,958	6,958	6,958
Firms	612	612	612	612	612	612	612	612
Anderson canon. corr. LM statistic	138.52***	10.84***	138.52***	10.84***	48.695***	1234.063***	48.695***	1234.063***
Cragg-Donald Wald F statistic	141.43	10.85	141.43	10.85	49.010	1530.044	49.010	1530.044

Notes: Dependent variables (DV) are shown at the top. The instruments used are EPU China (columns 1–4) and lagged banking performance (columns 5–8).

* p<0.1;

** p<0.05;

*** p<0.01.

Standard errors are in parentheses.

### 4.4. Tests of mechanisms

Our findings collectively present compelling evidence that uncertainty in the banking sector negatively impacts firms’ debt in Vietnam. In this particular subsection, our objective is to explore the underlying mechanisms by which banking uncertainty influences corporate debt, guided by four potential channels discussed in the literature review section. To systematically investigate these channels, we employ a two-step approach. In the first step, we analyze the impact of uncertainty on each mediator individually (including the cost of debt, trade credit, financial distress, and investment contractions). Subsequently, in the second step, we estimate the influence of these mediators on corporate debt. Many recent studies on finance and uncertainty employ this approach for channel tests with mediators [51, 52, 58–60].

To fulfill this objective, we employ specific metrics for each mediator. The debt financing cost of firms is gauged by the ratio of interest payments to total debt (*cost*). Assessing firms’ financial distress involves utilizing Altman’s Z-score, wherein larger scores indicate lower financial distress (*Zscore*). Trade credit is operationalized through two variables: (i) accounts payable scaled by total assets (*tc1*) and (ii) accounts payable scaled by the cost of goods sold (*tc2*), providing a robust depiction of the findings. Similarly, two alternative measures are employed for firm investments: the year-by-year change in capital expenditures (*capex1*) and the ratio of capital expenditures to total assets (*capex2*).

Table 9 provides an overview of the test results for the cost-of-debt channel. For conciseness, we omit reporting the results of all control variables. The estimates of the first step, utilizing the cost of debt as the dependent variable, incorporate standard firm-specific and macro control variables drawn from pertinent prior research [61–63]. In columns 1–2, the results indicate a positive and statistically significant effect of banking uncertainty on the cost of debt. Subsequently, columns 3–4 reveal a negative and statistically significant impact of the cost of debt on corporate debt. It is important to note that results for the other firm debt variable (*debt2*) in this set of tests and subsequent tests are qualitatively similar and omitted for conciseness. These findings suggest that heightened uncertainty increases the cost of debt, and such an escalation in the cost of financing prompts firms to adjust their capital structures by cutting the use of debt. Consequently, these results provide evidence of how banking uncertainty can diminish debt financing through the cost-of-debt channel.

**Table 9:** Tests for the cost of debt channel.

	(1)	(2)	(3)	(4)
	cost	cost	debt1	debt1
Cost			**-0.108** ***	**-0.109** ***
			**(0.011)**	**(0.011)**
aunc	**0.016** **		-0.136***	
	**(0.007)**		(0.012)	
func		**0.007** **		-0.047***
		**(0.003)**		(0.004)
lagged DV	-0.198***	-0.202***	-0.180***	-0.172***
	(0.007)	(0.007)	(0.020)	(0.020)
Macro controls	Yes	Yes	Yes	Yes
Firm controls	Yes	Yes	Yes	Yes
Observations	4,965	4,965	5,459	5,459
Firms	567	567	588	588
Instruments	118	118	120	120
AR(1) test	0.000	0.000	0.000	0.000
AR(2) test	0.419	0.453	0.101	0.340
Hansen test	0.160	0.175	0.450	0.460

Notes: Dependent variables (DV) are shown at the top.

** p<0.05;

*** p<0.01.

Standard errors are in parentheses.

Transitioning to the trade credit channel examinations as delineated in Table 10, our findings reveal a positive association between trade credit and uncertainty (columns 1–2 and 5–6), indicating that firms tend to augment their utilization of trade credit during periods of heightened banking uncertainty. This observation is derived after regressing a trade credit model with controls consistent with the existing literature [64, 65]. Subsequently, the remaining columns in Table 10 demonstrate that companies with greater trade credit tend to exhibit reduced interest-bearing debt, as evidenced by the significantly negative coefficients on the trade credit variables in the debt model. This suggests that trade credit is one of the economic mechanisms through which banking uncertainty drives firms’ financial structure. Our finding also complements the work of Zhang et al. [16] and Fan et al. [21], who assert that firms may transition from bank debt to trade credits in response to escalating uncertainty. This result also aligns with the earlier finding in the present study that banking uncertainty reduces the use of other forms of debt, such as corporate bonds, thereby strongly supporting the notion that short-term trade credit emerges as an alternative source of financing, substituting for traditional interest-bearing debts such as bank loans and corporate bonds.

**Table 10:** Tests for the trade credit channel.

	(1)	(2)	(3)	(4)	(5)	(6)	(7)	(8)
	tc1	tc1	debt1	debt1	tc2	tc2	debt1	debt1
tc1			**-0.077** **	**-0.079** **				
			**(0.033)**	**(0.032)**				
tc2							**-0.039** ***	**-0.040** ***
							**(0.008)**	**(0.008)**
aunc	**0.012** **		-0.159***		**0.129** ***		-0.165***	
	**(0.006)**		(0.011)		**(0.027)**		(0.011)	
func		**0.006** ***		-0.053***		**0.077** ***		-0.055***
		**(0.002)**		(0.004)		**(0.014)**		(0.004)
lagged DV	-0.062***	-0.061***	-0.041**	-0.035**	-0.034***	-0.036***	-0.053***	-0.046***
	(0.014)	(0.014)	(0.017)	(0.017)	(0.011)	(0.012)	(0.017)	(0.017)
Macro controls	Yes	Yes	Yes	Yes	Yes	Yes	Yes	Yes
Firm controls	Yes	Yes	Yes	Yes	Yes	Yes	Yes	Yes
Observations	6,969	6,969	6,969	6,969	6,963	6,963	6,967	6,967
Firms	623	623	623	623	623	623	623	623
Instruments	120	120	121	121	120	120	121	121
AR(1) test	0.000	0.000	0.000	0.000	0.000	0.000	0.000	0.000
AR(2) test	0.101	0.102	0.470	0.133	0.279	0.287	0.426	0.108
Hansen test	0.187	0.225	0.462	0.449	0.473	0.436	0.429	0.477

Notes: Dependent variables (DV) are shown at the top.

** p<0.05;

*** p<0.01.

Standard errors are in parentheses.

In columns 1–2 of Table 11, where the list of control variables is adopted based on the related literature [66, 67] to yield estimation results of financial distress, we observe a negative association between banking uncertainty and Z-score–a reverse measure of financial distress. This indicates that uncertainty leads to an escalation of firm financial distress risk. Simultaneously, the results in columns 3–4 of Table 11 demonstrate significantly negative coefficients for Z-score when regressing on firms’ debt, signifying that financial distress can augment corporate debt holdings. Considering that uncertainty increases firms’ financial distress, however, our findings do not support the notion that it is likely to decrease their debt holdings through the financial distress risk channel. This result diverges from Fan et al. [21], who document that higher oil price uncertainty may elevate a firm’s financial distress, thus cutting its corporate leverage.

**Table 11:** Tests for the financial distress risk channel.

	(1)	(2)	(3)	(4)
	Zscore	Zscore	debt1	debt1
Zscore			**-0.007** ***	**-0.007** ***
			**(0.001)**	**(0.001)**
aunc	**-1.073** ***		-0.144***	
	**(0.130)**		(0.011)	
func		**-0.372** ***		-0.049***
		**(0.045)**		(0.004)
lagged DV	0.149***	0.154***	-0.059***	-0.057***
	(0.016)	(0.016)	(0.017)	(0.017)
Macro controls	Yes	Yes	Yes	Yes
Firm controls	Yes	Yes	Yes	Yes
Observations	6,967	6,967	6,969	6,969
Firms	623	623	623	623
Instruments	117	117	121	121
AR(1) test	0.000	0.000	0.000	0.000
AR(2) test	0.601	0.554	0.521	0.178
Hansen test	0.117	0.115	0.475	0.463

Notes: Dependent variables (DV) are shown at the top.

*** p<0.01.

Standard errors are in parentheses

Finally, Table 12 provides results substantiating the significance of uncertainty shocks in propelling fluctuations in debt through the effects of investment. In our models of firm investment, we also incorporate control variables as recommended by the relevant literature [7, 8]. The results reveal a significantly negative impact of uncertainty on firm investment (columns 1–2 and 5–6), and a significantly positive impact of investment on corporate debt (columns 3–4 and 7–8). These findings indicate that banking uncertainty is likely to curtail firm investment, and this reduction in investment could, in turn, diminish firms’ reliance on debt. In summary, our results provide support for the investment reduction channel.

**Table 12:** Tests for the investment reduction channel.

	(1)	(2)	(3)	(4)	(5)	(6)	(7)	(8)
	capex1	capex1	debt1	debt1	capex2	capex2	debt1	debt1
capex1			**0.084** ***	**0.089** ***				
			**(0.017)**	**(0.017)**				
capex2							**0.198** ***	**0.190** ***
							**(0.018)**	**(0.018)**
aunc	**-0.016** **		-0.163***		**-0.025** ***		-0.158***	
	**(0.007)**		(0.012)		**(0.007)**		(0.011)	
func		**-0.009** ***		-0.053***		**-0.012** ***		-0.051***
		**(0.003)**		(0.004)		**(0.003)**		(0.003)
lagged DV	0.070***	0.072***	-0.062***	-0.056***	-0.181***	-0.184***	-0.074***	-0.065***
	(0.006)	(0.006)	(0.018)	(0.018)	(0.011)	(0.011)	(0.017)	(0.017)
Macro controls	Yes	Yes	Yes	Yes	Yes	Yes	Yes	Yes
Firm controls	Yes	Yes	Yes	Yes	Yes	Yes	Yes	Yes
Observations	6,504	6,504	6,504	6,504	6,969	6,969	6,969	6,969
Firms	619	619	619	619	623	623	623	623
Instruments	108	108	112	112	118	118	121	121
AR(1) test	0.000	0.000	0.000	0.000	0.000	0.000	0.000	0.000
AR(2) test	0.197	0.154	0.462	0.192	0.763	0.200	0.610	0.204
Hansen test	0.493	0.557	0.162	0.109	0.168	0.194	0.251	0.285

Notes: Dependent variables (DV) are shown at the top.

** p<0.05;

*** p<0.01.

Standard errors are in parentheses.

## 5. Further tests

### 5.1. Moderating role of bank relationship

Our finding thus far has shown that uncertainty in banking causes a decline in firms’ debt holdings in their capital structure. In this vein, an issue emerging from our finding is that if uncertainty arises from the banking industry affecting firms’ capital structures, it is natural to expect this impact to be stronger for firms maintaining a close relationship with banks. In other words, firms that rarely borrow from banks or do not rely heavily on banks will be less affected by a shock in the banking sector. In this part, we need to examine the impact of the bank-firm relationship on the link between banking uncertainty and corporate debt.

To execute this task, we proxy a bank-firm relationship (*bankfirm*) by determining whether a firm had a long-term loan contract in the previous year [16]. Subsequently, we augment the baseline model by incorporating an interaction between the bank-firm relationship variable and the uncertainty measure. The interaction variable serves as the primary explanatory factor gauging how the bank-firm relationship modulates the link between banking uncertainty and corporate leverage. Analyzing the estimation results presented in Table 13, a negative coefficient on the interaction—aligned with the sign of stand-alone uncertainty measures—suggests that the bank-firm relationship could intensify the adverse impact of uncertainty on corporate debt. Following a banking sector uncertainty shock, we posit that firms relying more heavily on banks would experience heightened susceptibility, leading them to potentially reduce their debt holdings. This finding is different from that of Zhang et al. [16], who point out that companies tend to reduce their financial leverage as economic policy uncertainty intensifies, and this effect is mitigated for firms having prior bank-firm connections thanks to bank support amid economic policy uncertainty. The disparity in our results may arise from the specific focus of our present research on banking uncertainty, resulting in a distinctive pattern compared to the general economic uncertainty identified by Zhang et al. [16].

**Table 13:** Moderating role of bank-firm relationship.

	(1)	(2)	(3)	(4)
	debt1	debt1	debt2	debt2
aunc	-0.136***		-0.245***	
	(0.013)		(0.023)	
aunc*bankfirm	-0.257***		-0.347***	
	(0.024)		(0.043)	
func		-0.051***		-0.100***
		(0.004)		(0.008)
func*bankfirm		-0.056***		-0.045***
		(0.008)		(0.017)
bankfirm	-0.108***	-0.044**	-0.238***	-0.221***
	(0.008)	(0.020)	(0.015)	(0.014)
lagged DV	-0.109***	-0.115***	-0.106***	-0.044**
	(0.023)	(0.029)	(0.023)	(0.021)
Macro controls	Yes	Yes	Yes	Yes
Firm controls	Yes	Yes	Yes	Yes
Observations	6,969	6,969	6,969	6,969
Firms	623	623	623	623
Instruments	120	120	120	120
AR(1) test	0.000	0.000	0.000	0.000
AR(2) test	0.578	0.577	0.558	0.502
Hansen test	0.117	0.290	0.118	0.146

Notes: Dependent variables (DV) are shown at the top.

** p<0.05;

*** p<0.01.

Standard errors are in parentheses.

### 5.2. Moderating role of macro shocks

Existing research underscores the pivotal role of financial institutions in facilitating firms’ access to finance during crises. Economic contractions, constraining access to preferred financing sources, can reshape corporate financial structure [68]. Furthermore, the literature consistently posits that uncertainty tends to surge in weaker economic conditions [6]. In times of crisis, heightened uncertainty and increased risk, coupled with declining expected returns, lead lenders and borrowers to exhibit reluctance in committing capital to investments, thereby influencing corporate debt.

Considering the pertinent factors outlined above, it is prudent to account for the potential combined impacts of crises and uncertainty on firms’ debt-taking decisions. Previous research has primarily emphasized the moderating roles of firm characteristics, overlooking the potential joint influence of uncertainty and other macro shocks on the financial structure of firms. The present study addresses this gap by employing an extended specification with interaction terms to examine changes in financial structure in response to banking uncertainty during crisis periods. The two crisis episodes encompass the global financial crisis (crisis = 1 for 2007–2009, and 0 otherwise) and the COVID-19 pandemic (covid = 1 for 2020–2021, and 0 otherwise). Concise estimation results with key variables of interest are presented in Table 14. The interaction term of interest has significant and negative coefficients in all columns. This result consistently reveals that the adverse effect of banking uncertainty on firm debt is strengthened amid periods with macro shocks. Our findings still hold no matter what bank-level shocks are considered to capture banking uncertainty. This result is consistent with the view that during macroeconomic shocks, financing costs increase [69], trade credit surges [70], and investment falls sharply [71]. These are all proven transmission mechanisms of banking uncertainty to debt financing, making the impact more significant during macroeconomic shocks.

**Table 14:** Moderating role of macro shocks.

	(1)	(2)	(3)	(4)	(5)	(6)	(7)	(8)
	debt1	debt1	debt2	debt2	debt1	debt1	debt2	debt2
aunc	-0.271***		-0.427***		-0.114***		-0.180***	
	(0.024)		(0.041)		(0.012)		(0.020)	
aunc*crisis	-0.113***		-0.175***					
	(0.013)		(0.023)					
func		-0.145***		-0.225***		-0.037***		-0.063***
		(0.017)		(0.031)		(0.004)		(0.007)
func*crisis		-0.035***		-0.053***				
		(0.006)		(0.012)				
aunc*covid					-0.187***		-0.369***	
					(0.020)		(0.039)	
func*covid						-0.169***		-0.326***
						(0.017)		(0.032)
crisis	-0.048***	-0.097***	-0.064***	-0.139***				
	(0.008)	(0.018)	(0.014)	(0.032)				
covid					-0.069***	-0.071***	-0.144***	-0.142***
					(0.010)	(0.010)	(0.016)	(0.016)
lagged DV	-0.042**	-0.045***	-0.051***	-0.045***	-0.081***	-0.079***	-0.081***	-0.077***
	(0.017)	(0.017)	(0.017)	(0.017)	(0.017)	(0.016)	(0.017)	(0.016)
Macro controls	Yes	Yes	Yes	Yes	Yes	Yes	Yes	Yes
Firm controls	Yes	Yes	Yes	Yes	Yes	Yes	Yes	Yes
Observations	6,969	6,969	6,969	6,969	6,969	6,969	6,969	6,969
Firms	623	623	623	623	623	623	623	623
Instruments	120	120	120	120	120	120	120	120
AR(1) test	0.000	0.000	0.000	0.000	0.000	0.000	0.000	0.000
AR(2) test	0.423	0.450	0.470	0.408	0.188	0.275	0.320	0.307
Hansen test	0.424	0.345	0.326	0.583	0.479	0.445	0.521	0.473

Notes: Dependent variables (DV) are shown at the top.

** p<0.05;

*** p<0.01.

Standard errors are in parentheses.

## 6. Conclusions

In this study, we employ the uncertainty index in the banking sector and utilize a panel data set covering Vietnamese listed firms spanning from 2007 to 2022 in the empirical analysis of GMM and fixed effect estimations to explore how firms adjust their debt holdings amid banking uncertainty. Our key findings are as follows. First, we observe that banking uncertainty negatively impacts firm debt. This effect holds across various debt maturities and sources, with the most significant fluctuations noted in bank debt. Our findings withstand robustness checks involving alternative measures of firms’ debt, different proxies for banking shocks to represent uncertainty, various econometric models, and addressing potential endogeneity issues. Second, we provide insights into the underlying mechanisms by which banking uncertainty shapes corporate financial structure, encompassing increased financial costs, substitution of trade credit, and contractions in firm investment. Third, we identify the moderating role of both firm-specific and macro characteristics, which can modify the adverse effects of banking uncertainty on firms’ debt utilization. We observe that corporate debt exhibits more pronounced reactions to banking uncertainty for firms with closer ties to banks or during macroeconomic shocks, as evidenced by the global financial crisis and the COVID-19 pandemic.

This paper carries several implications. The results obtained can offer valuable insights for policymakers in crafting effective strategies to alleviate the adverse consequences stemming from banking uncertainty, particularly in situations where a shortfall in firms’ debt, especially from the source of bank debt, could hurt enterprises’ operation, thereby impacting overall enterprise development and economic growth. This holds heightened significance when banks serve as a crucial financing channel during businesses’ need for external funding and when the impact is notably pronounced in firms with established lending relationships with banks and during periods of economic turmoil. Under this line, central banks should provide clear and consistent communication about monetary policy and regulatory changes to reduce uncertainty. Next, support in terms of financing costs, alternative funding sources, or improved policies to expand investment opportunities may be suggestions in the context of increased uncertainty. For example, governments can offer loans at subsidized interest rates to businesses and provide temporary tax relief or deferrals to firms to reduce their financial burden, as was implemented in various countries during the pandemic. Additionally, our findings provide crucial guidance for firms’ managers and investors, aiding them in formulating appropriate financing and investment decisions. For instance, firms can adopt strategies to diversify their funding sources by shifting toward trade credit or equity financing. This reduces their dependence on traditional bank loans and enhances their financial stability.

We acknowledge that this study focuses not on the intensity of the total mechanism effects, but rather on confirming the underlying mechanisms through the required intermediary steps. Additionally, it is important to note that an increased cost of debt impacts both financial distress—due to the higher likelihood of returns on assets falling below the interest rate—and investment decisions, as a higher internal rate of return is necessary for investments to be deemed viable. Therefore, a comprehensive model that incorporates more complex mechanisms, including the effects of serial mediators, particularly the impact of the cost of debt on other mediators, warrants careful analysis in future research.
